# Novel Antagonists of Heparin Binding Growth Factors

**DOI:** 10.18632/oncotarget.645

**Published:** 2012-09-04

**Authors:** Fabiola Cecchi, Donald P. Bottaro

**Affiliations:** Urologic Oncology Branch, National Cancer Institute, Bethesda, MD, USA; Urologic Oncology Branch, National Cancer Institute, Bethesda, MD, USA

Heparin and heparan sulfate proteoglycans (HSPGs) are structurally diverse biopolymers that modulate many important protein-protein interactions. As ubiquitous components of extracellular matrices, the extended conformation, inherent complexity and high negative charge density of HSPGs enhance tissue integrity and provide selective yet substantial protein binding capacity [1]. Hepatocyte growth factor (HGF) and vascular endothelial cell growth factor (VEGF) regulate development and homeostasis, and drive tumorigenesis, tumor angiogenesis and metastasis in many forms of cancer. Both proteins signal by binding to receptor tyrosine kinases (RTKs) and HSPGs on target cell surfaces. Their respective RTKs also directly bind HSPGs, enabling the formation of ternary ligand-HSPG-RTK complexes with enhanced stability and signaling capacity [1, 2].

In a recent report, Cecchi et al. compared the HSPG binding sites on HGF and VEGF and found that critical basic HSPG binding residues provided similar surface charge distributions without underlying sequence or structural similarity, suggestive of convergent evolutionary adaptation to the common binding partner [3]. Prior studies to assess the impact of HSPG on HGF or VEGF signaling using alanine substitutions at HSPG binding residue positions showed some functional perturbation, but not to an extent consistent with the importance of HSPG in VEGF and HGF signaling gleaned from a variety of other studies. To reconcile this apparent discrepancy, Cecchi et al. used another approach to assess the impact of HSPG binding, wherein three opposite charge substitutions were combined in these sites in the HGF isoform NK1 (NK1 3S) or the VEGF isoform VEGF165 (VEGF 3S), with the goal of repelling HSPG from the ligand-RTK complex [3]. Remarkably, the substituted proteins were devoid of biological activity but retained native RTK binding affinity, and thus competitively antagonized their respective signaling pathways in normal and tumor-derived cells *in vitro* and *in vivo* [3].

NK1 3S and VEGF 3S antagonism of HGF and VEGF signaling, respectively, occurs through the combination of charge-based repulsion of HSPG and competitive displacement of the endogenous ligand from its RTK. Interestingly, NK1 3S retains optimal HGF receptor (Met) binding not only because its Met binding residues are unaltered, but also because HSPG-HGF binds more tightly to Met than HGF alone, suggesting that the acidic substitutions in NK1 3S mimic bound HSPG in this regard [3]. They fail to mimic bound HSPG, however, for the purpose of Met activation. Only HSPG polymers capable of binding multiple HGF molecules and enhancing HGF:HGF aggregation enabled Met signaling in HSPG-negative cells [3]. Together with prior studies, these results suggest that HSPG promotes HGF-Met complex clustering and, in turn, Met-Met interactions needed for kinase activation.

In the VEGF receptor KDR, VEGF binds to IgG-like domains (D) 1-3; D2 contains primary contacts and D1 and D3 contribute to binding affinity and specificity [4]. HSPG functions similarly in the activation of Met and KDR. Like NK1, HSPG is required for high affinity binding of VEGF165 to KDR ectopically expressed in HSPG-negative cells [5]. Similar to Met [6] and fibroblast growth factor (FGF) receptors [2], KDR also interacts directly with HSPG, through at least one site located between D6 and D7 [5]. As shown for other family members and related receptors [7], a series of binding events may promote and incrementally stabilize HSPG-VEGF-KDR aggregates capable of signaling: VEGF-HSPG binding to KDR D2 stabilizes weaker VEGF-D1 and -D3 interactions, with additional stability gained through HSPG-VEGF-KDR bridging at D6/D7. These events, in turn, induce and/or stabilize conformational changes that enable homotypic D7 interactions and finally, TK domain interaction and transactivation [4]. VEGF 3S binds KDR at D2, but by repelling HSPG from the complex, is likely to destabilize some subsequent weaker interactions and ultimately, conformation changes leading to TK activation.

Structural and functional studies of ligand-RTK interactions over the last decade highlight the importance of multiple binding events and associated conformational changes in RTK ectodomains that are required for kinase activation. These events vary in strength, and even weak interactions appear to provide necessary increments of increased stability to a signal transduction process whose complexity we are only beginning to appreciate. The acquisition of competitive antagonism by 3S forms of HGF and VEGF exposes the susceptibility of HSPG-facilitated events to selective disruption, and functionally defines the importance of HSPG in the ternary HS-ligand-RTK complex in normal and oncogenic signaling [3]. By tethering multiple long chain HS polymers, HSPGs stabilize ligand-RTK binding events by binding to both; beyond this, HSPGs have the potential to stabilize the aggregation of ligand-RTK complexes that is likely to accompany larger scale processes, such as endocytosis and endosomal sorting, that coincide with mitogenic and motogenic signaling and that have been shown to significantly influence the nature and magnitude of these cellular responses [8]. Future studies of HSPGs in sustaining endosomal RTK signaling should address, for example, whether HSPG fragmentation in early endosomes negatively regulates RTK signaling, and whether HSPG association affects the fate of ligand-RTK complexes to recycling vs. lysosomal endpoints, as shown for FGF-FGFR [9], and likely to be true for HGF-Met [10]. Further defining the role of HSPG in these large scale signaling processes should help identify cancer-associated aberrations and new opportunities for targeting this system to control disease progression.
